# Are commercial probiotics and prebiotics effective in the treatment and prevention of honeybee nosemosis C?

**DOI:** 10.1007/s00436-015-4761-z

**Published:** 2015-10-06

**Authors:** Aneta A. Ptaszyńska, Grzegorz Borsuk, Agnieszka Zdybicka-Barabas, Małgorzata Cytryńska, Wanda Małek

**Affiliations:** Department of Botany and Mycology, Institute of Biology and Biochemistry, Faculty of Biology and Biotechnology, Maria Curie-Skłodowska University, 19 Akademicka st., 20-033 Lublin, Poland; Department of Biological Basis of Animal Production, Faculty of Biology and Animal Breeding, University of Life Sciences, 13 Akademicka st., 20-950 Lublin, Poland; Department of Immunobiology, Institute of Biology, Maria Curie-Skłodowska University, 19 Akademicka st., 20-033 Lublin, Poland; Department of Genetics and Microbiology, Maria Curie-Skłodowska University, 19 Akademicka st., 20-033 Lublin, Poland

**Keywords:** *Apis mellifera* survival, *Nosema ceranae*, Phenoloxidase activity, Probiotic, Prebiotic, *Lactobacillus rhamnosus*, inulin

## Abstract

The study was conducted to investigate the effect of *Lactobacillus rhamnosus* (a commercial probiotic) and inulin (a prebiotic) on the survival rates of honeybees infected and uninfected with *Nosema ceranae*, the level of phenoloxidase (PO) activity, the course of nosemosis, and the effect on the prevention of nosemosis development in bees. The cells of *L. rhamnosus* exhibited a high rate of survival in 56.56 % sugar syrup, which was used to feed the honeybees. Surprisingly, honeybees fed with sugar syrup supplemented with a commercial probiotic and a probiotic + prebiotic were more susceptible to *N. ceranae* infection, and their lifespan was much shorter. The number of microsporidian spores in the honeybees fed for 9 days prior to *N. ceranae* infection with a sugar syrup supplemented with a commercial probiotic was 25 times higher (970 million spores per one honeybee) than in a control group fed with pure sucrose syrup (38 million spores per one honeybee). PO activity reached its highest level in the hemolymph of this honeybee control group uninfected with *N. ceranae*. The addition of probiotics or both probiotics and prebiotics to the food of uninfected bees led to the ~2-fold decrease in the PO activity. The infection of honeybees with *N. ceranae* accompanied an almost 20-fold decrease in the PO level. The inulin supplemented solely at a concentration of 2 μg/mL was the only administrated factor which did not significantly affect honeybees’ survival, the PO activity, or the nosemosis infection level. In conclusion, the supplementation of honeybees’ diet with improperly selected probiotics or both probiotics and prebiotics does not prevent nosemosis development, can de-regulate insect immune systems, and may significantly increase bee mortality.

## Introduction

All members of the Animalia kingdom, including humans, have helpful symbiotic microbiota which are extremely important for the proper functioning of the gastrointestinal tract. These symbiotic microorganisms are responsible for the fermentation of carbohydrates as well as the production of some vitamins and amino acids that their hosts need. Furthermore, gut microbiota, through the “barrier effect,” prevent pathogenic microorganisms from colonizing the gastrointestinal tract. In particular, lactic acid bacteria (LAB) prove to be important inhabitants of animal and human intestinal tracts as they have a multifaceted, antimicrobial potential, mainly because of their ability to synthesize lactic acid, short-chain, volatile fatty-acid, and bacteriocin-like molecules (Jack et al. 1995; Wilson et al. 2005; Audisio et al. 2011). Lactic acid bacteria are usually considered probiotics, i.e., viable microorganisms that provide health benefits to their hosts (Schlundt 2012). Probiotics are helpful in the treatment of several human illnesses, including diarrhea, allergies, obesity, lactose intolerance, inflammation, *Helicobacter pylori* infections, necrotizing enterocolitis (NEC), eczema, and many others. Successful marketing strategies and the popularization of probiotics have led to these products being commonly used as dietary supplements. Also, prebiotics which are non-digestible fiber compounds cause specific changes, both to the composition and/or activity of gastrointestinal microflora, and confer benefits upon their hosts’ well-being and health (Roberfroid 2007). One such prebiotic is inulin, a linear chain of (2-1)-linked β-d-fructosyl units, which selectively promotes the growth and activity of bacteria from the genus *Bifidobacterium* that are beneficial for human and animal health (Cummings et al. 2001; Urías-Silvas et al. 2008).

Probiotics and prebiotics are recommended to be added not only to the human diet but also into the forage of different vertebrates as well as invertebrates (e.g., Weese and Arroyo 2003; Patterson and Burkholder 2003; Ötleş 2013; Verlinden et al. 2006; Bagheri et al. 2008; Talpur et al. 2012). Certainly, the most beneficial effect is observed when organisms are provided with probiotics that had been previously isolated from themselves. However, LAB isolated from humans were found to have been used with positive results in the husbandry of terrestrial animals and for agricultural health management; e.g., *Lactobacillus rhamnosus* and *Lactobacillus bulgaricus* were indicated to be protective against opportunistic pathogens in fish farming (Nikoskelainen et al. 2001; Ötleş 2013). Also, in beekeeping management, there are commercial diet supplements which contain probiotics and/or prebiotics. One such supplement recommended for the feeding of honeybees and other animals contains bacteria such as *Lactobacillus casei*, *Lactobacillus plantarum*, *Rhodopseudomonas palustris*, and yeast *Saccharomyces cerevisiae*. A further example, in addition to lactic acid bacteria (*Lactobacillus acidophilus* or *L. casei*) and *Bifidobacterium lactis*, also comprises prebiotics (Pătruică and Mot 2012; Pătruică and Hutu 2013; Andrearczyk et al. 2014).

In honeybee guts and crops, several symbiotic bacteria were reported (Engel et al. 2012; Corby-Harris et al. 2014). They mainly belong to the *Lactobacillus* and *Bifidobacterium* genera and to the Acetobacteraceae family. Additionally, two other probiotic bacterial species, i.e., *Gilliamella apicola* and *Snodgrasella alvi*, were identified in honeybee alimentary tracts (Engel et al. 2012; Corby-Harris et al. 2014).

*Nosema ceranae*, the causative agent of nosemosis C, is an obligate, intercellular pathogen which completes its life cycle in honeybee intestines (Wittner and Weiss 1999; Ptaszyńska et al. 2014; Roberts et al. 2015). *N. ceranae* suppresses immune responses in honeybees (Antúnez et al. 2009; Chaimanee et al. 2012), causing a degeneration of gut epithelial cells (Higes et al. 2007; Dussaubat et al. 2012), a shortening of bee lifespans (Paxton et al. 2007; Higes et al. 2007; Dussaubat et al. 2012), and finally leading to a depletion of honeybee colonies. Insects defend themselves against pathogen infections by cellular immunity and humoral immune responses. These processes such as phagocytosis and encapsulation, in connection with melanization, play an important role in the cellular response. Phenoloxidase (PO) lysozyme and antimicrobial peptides such as abaecin, apidaecin, defensin, and hymeoptaecin are humoral factors essential for the antimicrobial defense of honeybees (Schmid-Hempel 2003; Evans et al. 2006; Cerenius et al. 2008).

Honeybees are very important pollinators which strongly influence the genomic diversity of the plant community; hence, their role in shaping the ecosystem can hardly be overestimated (Bradbear 2009). Currently, there are only a few articles concerning the effect of commercial probiotics and prebiotics on honeybee health. Some data have shown that commercial probiotics increase honeybee mortality, whereas others suggest that the administration of probiotics and prebiotics has an excellent effect on the growth of bee colonies and increases honey production (Pătruică and Mot 2012; Pătruică and Hutu 2013; Andrearczyk et al. 2014). Therefore, we decided to study the effect on honeybee health of *L. rhamnosus*, which plays a predominant role in the probiotics market (Douillard et al. 2013), and of inulin, a well-known prebiotic, (Slavin 2013), by analyzing PO activity, as well as the role of these supplements on the treatment and the prevention of the nosemosis in honeybees.

## Material and methods

### Animals, culture conditions and *N. ceranae* infection

Honeybees, *Apis mellifera carnica*, were maintained with standard beekeeping management methods in the university apiary (University of Life Sciences in Lublin, Poland). Honeybees were collected between the end of May 2014 and August of the same year. Although no permission is needed to administer experiments on insects, our research was planned in a way that reduced the number of honeybees to the minimum necessary for the proper conduction of these experiments. To obtain 1-day-old healthy honeybees, combs with brood originating from one queen bee were transferred, on the 20th day of bee development, to an air-conditioned chamber and kept at a constant temperature of 35 °C and at a humidity of 60 %. After emerging, honeybees were kept under laboratory conditions, in complete darkness (30 °C; *H* = 65 %) in wooden cages, occupied by 40 specimens.

In all experiments, honeybees were fed with a daily prepared 56.6 % sugar-water syrup (1:1; *w/v*) supplemented with commercial probiotics and/or prebiotics. The control honeybees were fed with a pure sugar-water syrup. Doses of the commercial probiotics and prebiotics used in experiments, i.e., 3750 CFU/syrup mL (group L2) and 2 μg/syrup mL (group In), respectively, were estimated on the basis of the manufacturer’s advice concerning a daily dosage of these supplements, taking 160 mg as an average honeybee weight. The average weight of honeybees was established after weighing 50 randomly chosen specimens of those being used in the experiments and was estimated at 157.6 mg.

To induce nosemosis, the honeybees were inoculated with a fresh solution containing 4 million *N. ceranae* spores/mL, in the amount of 8 μL per honeybee, according to the methodology described by Forsgren and Fries (2010). The spore inoculums were prepared from the ventriculi of naturally infected honeybees directly before experiments (Fries et al. 2013).

**Fig. 1:**
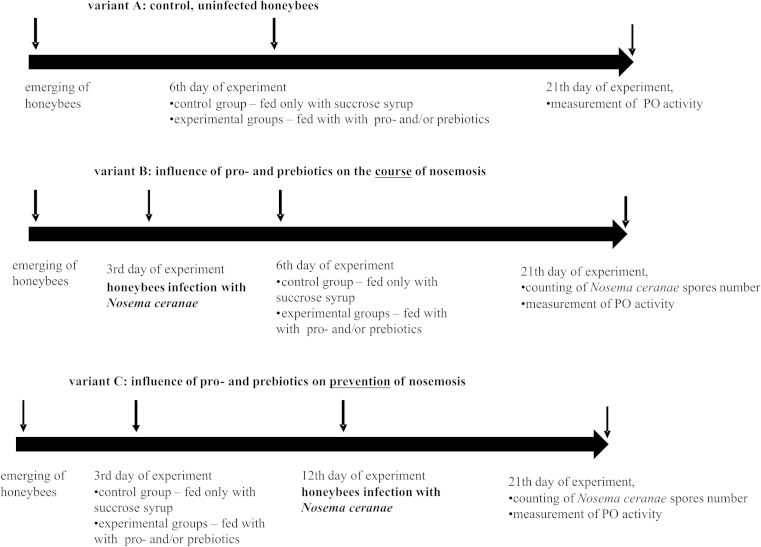
The scheme of administered experiments analyzing the effect of probiotics and prebiotics on the survival of honeybees uninfected and infected with *Nosema ceranae*

Emerging honeybees were divided randomly into three variants, “A,” “B,” and “C” (Fig. 1) with 36 cages in each. Honeybees in variant A served as a control and were not infected with *N. ceranae* (Fig. 1). In variant B, to check whether the supplementation of honeybee diets with commercial probiotic and/or prebiotic does influence the course of nosemosis, honeybees were *N. ceranae*-infected on the third day after emerging. Following this, from the sixth day until the end of the experiment, they were fed with a sugar-water syrup, containing commercial probiotic and/or prebiotic (Fig. 1). In variant C, to check whether the supplementation of honeybee diets with commercial probiotic and/or prebiotic does protect a host against nosemosis, honeybees from the third day after emerging until the end of the experiment were fed with a sugar-water syrup supplemented with probiotics and/or prebiotics, and after nine days of diet supplementation, these bees were infected with *N. ceranae* (Fig. 1).

Uninfected and *N. ceranae*-infected honeybees from variants A, B, and C were divided into six feeding groups, i.e., (1) SS; (2) L1; (3) L2; (4) In; (5) L1 + In; and (6) L2 + In. Concentrations of commercial probiotic and/or prebiotic among these groups were as follows: SS (control, pure sucrose syrup), L1 (1250 of *Lactobacillus* CFU/syrup mL, Biomed-Lublin, Poland), L2 (3750 of *Lactobacillus* CFU/syrup mL, Biomed-Lublin, Poland), In (inulin 2 μg/syrup mL, Frutafit^®^ IQ, Orafti, Belgium).

In all experiments, dead bees were counted every day, and the volume of eaten sugar syrup was estimated. Additionally, at the end of the experiments, the number of *N. ceranae* spores was counted and hemolymph PO activity was estimated.

### Estimation of the nosemosis level

Samples were prepared from every group in two repeats to count *N. ceranae* spores. For one sample, ten honeybee abdomens were grounded in 10 mL of sterile, distilled water, and the number of *Nosema* spores was counted according to Fries et al. (2013) and Hornitzky (2008) using a hemocytometer and Olympus BX61 light microscope. Furthermore, each sample was observed under bright field and differential interference contrast (DIC) to a proper differentiation of *N. ceranae* spores from other remains present in honeybee homogenates.

### Isolation of total DNA from honeybees and molecular detection of *N. ceranae*

Total DNA from uninfected and *N. ceranae*-infected *A. mellifera carnica* was isolated using the DNeasy Blood and Tissue Kit (Qiagen) according to the manufacturer’s instruction. To identify *N. ceranae*, DNA in the investigated samples using duplex PCR was conducted with 321-APIS and 218-MITOC primers (Martín-Hernández et al. 2007) in a 25-μL reaction mixture of the Qiagen Taq PCR Core Kit (Qiagen Inc.) containing 2.5 μL PCR buffer with 5 μL Q solution, 0.1 mM dNTP mixture, 0.7 U Taq DNA polymerase, 0.2 μM of each forward and reverse primers, approximately 0.15 μg of DNA template, and ddH_2_O to a final reaction volume of 25 μL. For DNA amplification, the following PCR cycling conditions were used: 1 min at 94 °C, 1 min at 61.8 °C, and 1 min at 72 °C, repeated for 30 cycles, and 10 min at 72 °C.

### The survival of *L. rhamnosus* (a commercial probiotic) in sugar syrup

The bacteria of the genus *Lactobacillus* used as the commercial probiotic were added to the number of 1250 and 3750 bacterial cells to 1 mL of 56.6 % sugar syrup. Resulting bacterial suspensions were left at 30 °C and at a humidity of 60 % to check the bacteria survival during their administration to the honeybees. After 1 min, and subsequently after 2, 3, 4, 6, 8, 10, 12, 14, 16, 18, 20, 22, 24, 26, 28, 38, 40, 48, and 96 h, the titer of the bacteria was determined by plating them on an MRS agar medium and incubating them for 24–48 h, at 37 °C, in anaerobic conditions. Ten colonies were then randomly selected to verify the taxonomic position of the cultured bacteria, on the basis of API^®^ CH50 strips (bioMérieux Clinical Diagnostics).

### Honeybee hemolymph collection

Hemolymph from ten individuals was collected in each experimental group in sterile-chilled Eppendorf tubes. The hemolymph was used to measure PO activity after the removal of hemocytes (Phenoloxidase (PO) activity assay section). For this purpose, first, the hemolymph was centrifuged at 4 °C at 200×*g* for 5 min, and next, the supernatant was centrifuged at 20,000×*g* for 15 min. After centrifugation, pooled supernatants were stored at −20 °C until used for PO activity measurement.

### PO activity assay

PO activity was determined in pooled hemolymph samples, according to a modified method, previously described by Park et al. 2005; Zdybicka-Barabas and Cytryńska 2010; Andrejko et al. 2014; Zdybicka-Barabas et al. 2014. Two microliters of the hemolymph, twice diluted in tris-buffered saline (TBS) (50 mM Tris–HCl pH 6.8, 1 mM NaCl), was combined with 18 μL of TBS, containing 5 mM CaCl_2_ in the wells of a 96-well plate (to a final sample volume of 20 μL). After 20 min of incubation at room temperature, 180 μL of 2 mM L-dihydroxyphenylalanine (L-DOPA) in 50 mM sodium phosphate, pH 6.5, was added. PO activity was determined spectrophotometrically, on the basis of the amount of melanin formed (absorbance at 490 nm) over 60 min, at 2-min intervals, using a microtiter plate reader (Bio-Rad Laboratories, Hercules, CA, USA). The PO activity was determined in three independent experiments, in triplicate, for each hemolymph sample.

### Statistical analysis

The SAS software (2002–2003) employing the ANOVA (a group and a variant effects were the experimental factors) and the Tukey’s honestly significant difference (HSD) test (SAS Institute 2002–2003) were used to prepare statistical analysis of the data obtained.

## Results and discussion

The survival of honeybees depends on their successful defense against different microbial parasites. Indigenous gut bacterial flora with the dominant role of lactic acid bacteria plays an important role in the protection of bees and other insects against colonization by pathogens and in the control of the growth of undesirable microorganisms (Jack et al. 1995; Wilson et al. 2005; Audisio et al. 2011).

The research was conducted to investigate the effect of *L. rhamnosus*, an important commercial probiotic, and of inulin, a widely known prebiotic, on the survival rate of honeybees, infected and uninfected with *N. ceranae*, to investigate the level of PO activity in the hemolymph of insects, and, furthermore, to analyze the role of the commonly used probiotics and prebiotics in the protection of bees against nosemosis C (Fig. 1).

**Fig. 2:**
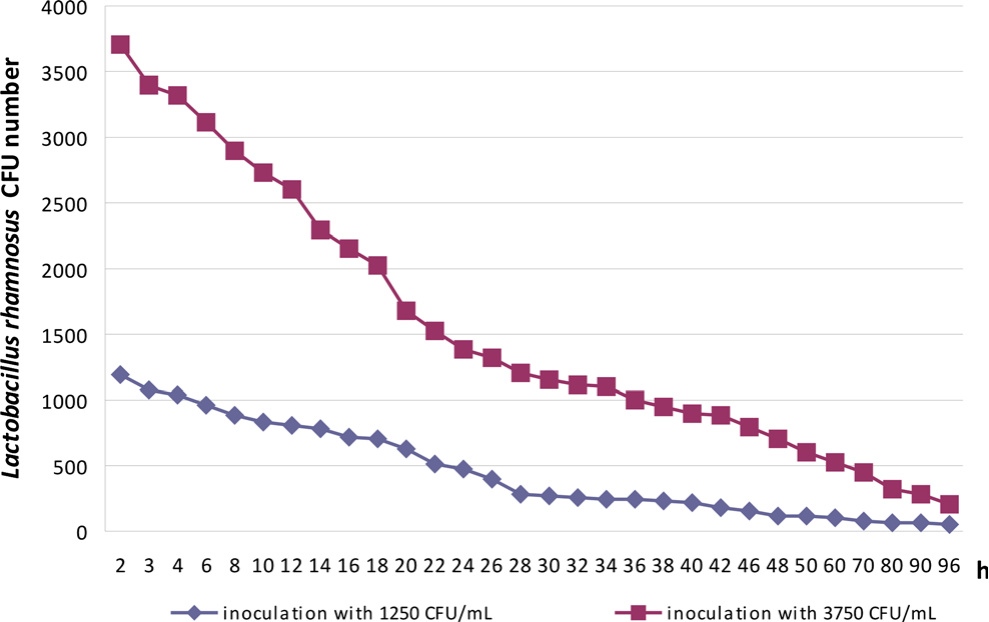
The survival of *Lactobacillus rhamnosus* during 96 h of incubation, in a 56.6 % sugar syrup, at 30 °C

The question posed initially concerned that of *L. rhamnosus* survival in 56.56 % sugar syrup used for honeybee feeding (Fig. 2). It is well known that sucrose, at high concentrations, induces osmotic stress in bacterial cells, connected with the loss of water from both membrane and proteins (Beney and Gervais 2001; Tymczyszyn et al. 2007; Randazzo et al. 2013), although at low concentrations, it becomes osmoprotectant. Lactobacilli survived in a 56.65 % sugar syrup used for honeybee feeding, for the studied period of time and even after 96 h of incubation. Under these conditions, viable and culturable bacterial cells were found after being plated on an MRS agar medium (Fig. 2). The bacteria grown on the MRS agar medium and selected randomly for API^®^ CH50 strip (bioMérieux Clinical Diagnostics) analysis exhibited the same fermentation profile of the 49 carbohydrates as the commercial strain of *L. rhamnosus* used in these experiments as the probiotic (data not presented).

**Fig. 3:**
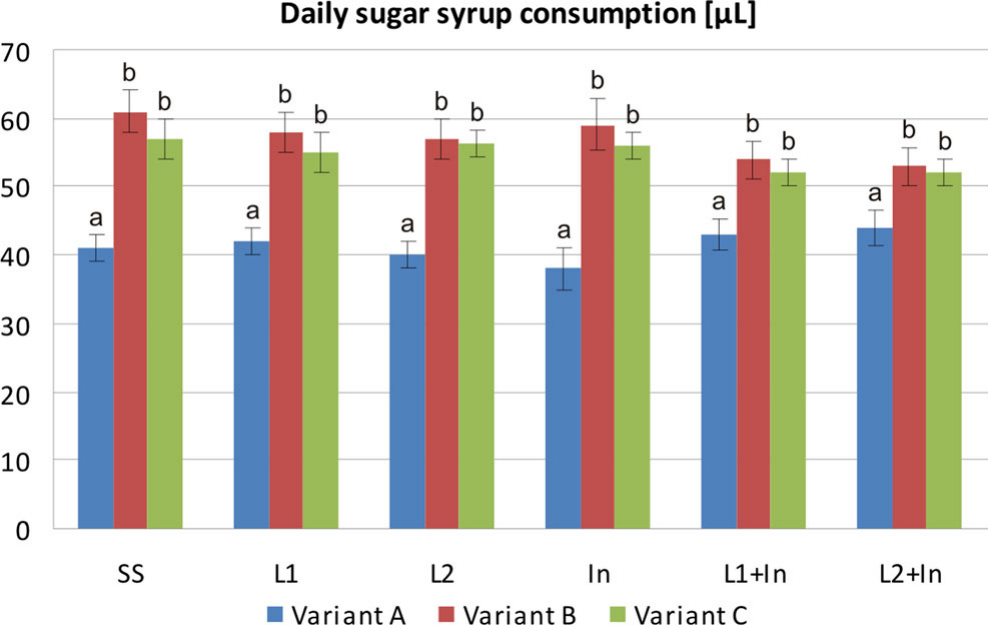
Consumption of sugar syrup supplemented with commercial probiotic and prebiotic by honeybees uninfected and infected with *N. ceranae*. Variants A, B, and C according to Fig. 1. Supplementation among the groups: SS (pure sugar syrup); L1 (1.25 × 10^3^ of *L. rhamnosus* CFU per 1 mL of sugar syrup); L2 (3.75 × 10^3^ of *L. rhamnosus* CFU per 1 mL of sugar syrup); In (2 μg of inulin per 1 mL of sugar syrup). *Error bars* represent standard deviations of data with lowercase letters indicating significant differences (*p* < 0.05)

Earlier data indicated that elevated levels of infection with pathogens may severely interfere with honeybees’ ability to absorb nutrients (Malone and Gatehouse 1998; Naug and Gibbs 2009; Mayack and Naug 2009; Martín-Hernández et al. 2011; Mayack and Naug 2013; Ptaszyńska et al. 2013; Ptaszyńska et al. 2014). These findings were also confirmed in the present study. It was found that a single uninfected honeybee consumed ~41 μL (±3.0) of the sugar syrup during a 24-h period, without any significant differences among the experimental groups (Fig. 3, variant A), while *N. ceranae*-infected honeybees consumed more sugar syrup, i.e., ~56 μL (±4.5) per bee, over 24 h (variants B and C, all studied groups). There are two possible explanations for nutritional and energy demands of honeybees infected with *Nosema* spp. being higher than those of uninfected insects. Firstly, parasitic microsporidia draw energy from the host for their own metabolic and reproductive needs. Secondly, honeybees infected with pathogens expend additional energy for mounting an immunological response, which is known to be an energy-expensive process (Schmid-Hempel 2003; Mayack and Naug 2009; Martín-Hernández et al. 2011; Borsuk et al. 2013; Naug 2014).

**Fig. 4:**
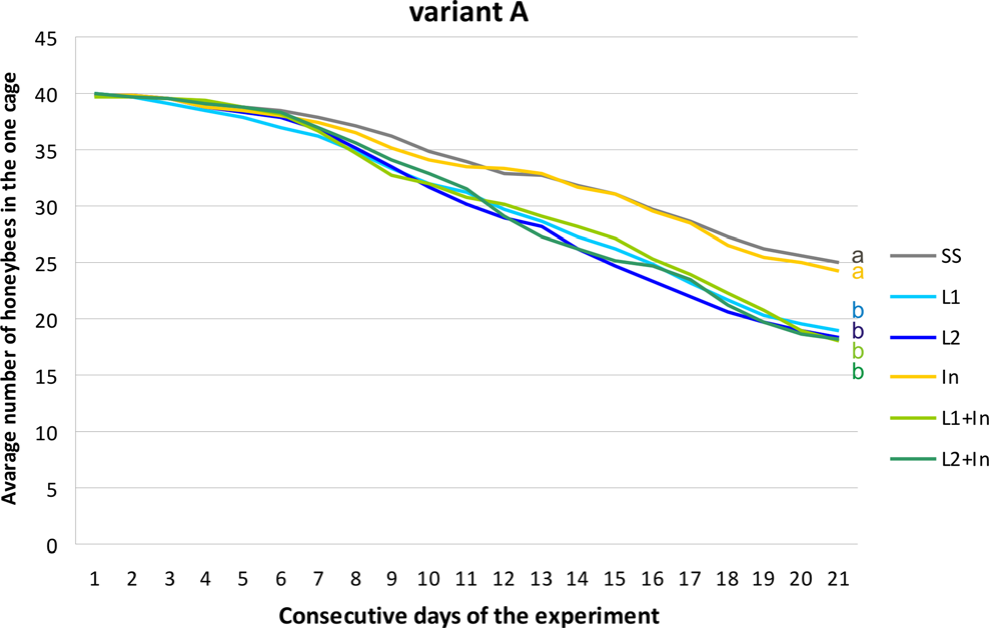
The survival of uninfected honeybees fed with commercial probiotics and prebiotics. Variant A according to Fig. 1. Supplementation among the groups: SS (pure sugar syrup); L1 (1.25 × 10^3^ of *L. rhamnosus* CFU per 1 mL of sugar syrup); L2 (3.75 × 10^3^ of *L. rhamnosus* CFU per 1 mL of sugar syrup); In1 (inulin 2 μg per 1 mL of sugar syrup). *Lowercase letters* indicate the differences significant for the comparison between variants *p* < 0.05

**Fig. 5:**
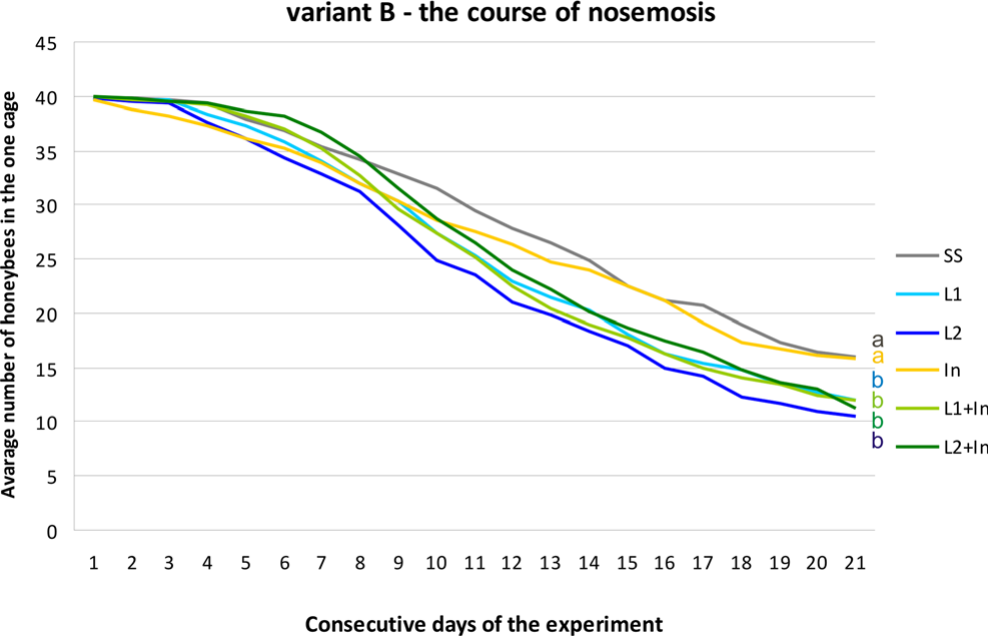
The survival of *N. ceranae*-infected honeybees fed with commercial probiotics and prebiotics. Variant B according to Fig. 1. Supplementation among the groups: SS (pure sugar syrup); L1 (1.25 × 10^3^ of *L. rhamnosus* CFU per 1 mL of sugar syrup); L2 (3.75 × 10^3^ of *L. rhamnosus* CFU per 1 mL of sugar syrup); In1 (inulin 2 μg per 1 mL of sugar syrup). *Lowercase letters* indicate the differences significant for the comparison between variants *p* < 0.05

**Fig. 6:**
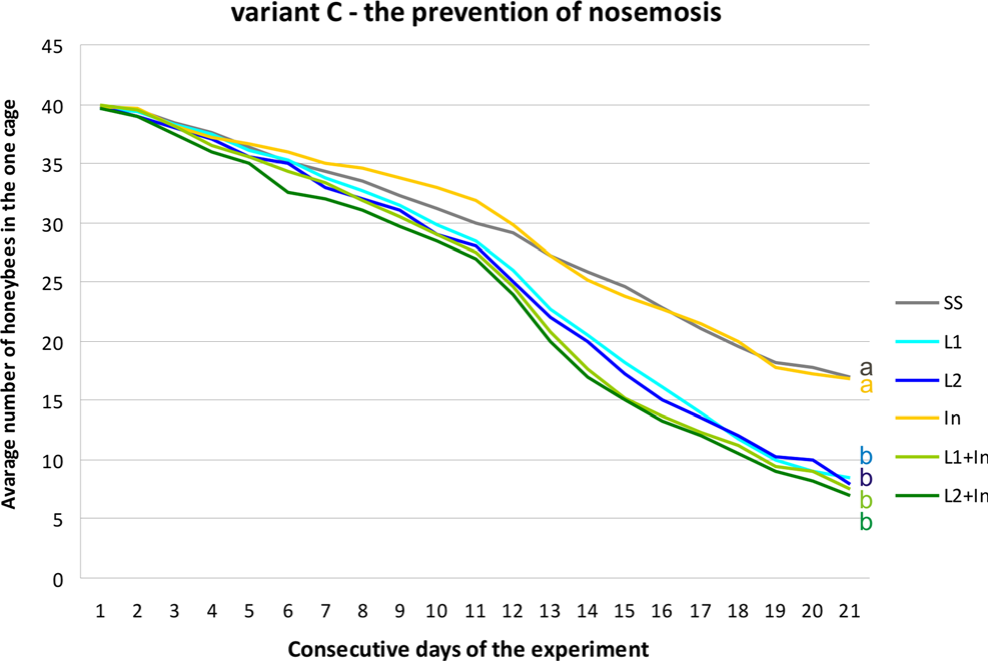
The survival of *N. ceranae*-infected honeybees fed with commercial probiotics and prebiotics. Variant C according to Fig. 1. Supplementation among the groups: SS (pure sugar syrup); L1 (1.25 × 10^3^ of *L. rhamnosus* CFU per 1 mL of sugar syrup); L2 (3.75 × 10^3^ of *L. rhamnosus* CFU per 1 mL of sugar syrup); In1 (inulin 2 μg per 1 mL of sugar syrup). *Lowercase letters* indicate the differences significant for the comparison between variants *p* < 0.05

In beekeeping management, several methods have been used to control infections caused by *Nosema* spp. In addition to good husbandry and good cultural conditions, nosemosis is traditionally controlled by heat treatment, fumigation, and, occasionally, by the administration of fumagillin (Porrini et al. 2010; Fries et al. 2013; Damiani et al. 2014; Strachecka et al. 2014). Live lactobacilli of the species *L. rhamnosus* (probiotic) with the documented inhibitory effects upon different pathogens (Ajitha et al. 2004) were analyzed in this investigation as a possible alternative to antimicrosporidian and prophylactic agents, supporting the natural defense mechanisms in honeybees. Surprisingly, supplementing honeybee diets solely with commercial probiotic (*L. rhamnosus*) and simultaneously with probiotic and prebiotic (inulin) increased mortality levels in both the *N. ceranae*-infected and uninfected honeybees. Inulin, at the concentration of 2 μg/mL, was the only administered factor which did not affect the honeybee survival rate in both group, i.e., uninfected and infected with *N. ceranae* (Figs. 4, 5, and 6). Martín-Hernández et al. (2011) explained the increased mortality of *Nosema* spp.-infected bees as energetic stress which may lead to a lack of thermoregulatory capacity and a higher rate of trophallaxis, leading to the increased spread of parasites and an increase in the bees’ mortality. Malnutrition connected with a nosemosis gut infection can further accelerate honeybee mortality (Mayack and Naug 2013; Ptaszyńska et al. 2014). As found in this study, the lower survival rate of bees fed with a sugar syrup containing probiotics, in comparison to control insects fed only with a sugar syrup, may be due to the competition for nutrients and energy resources between commercial probiotic bacteria and their host. In uninfected honeybees fed with a pure sucrose syrup, the mortality was established as 0.85 (±0.48) specimens daily per cage (Fig. 4, SS group). The mortality of *N. ceranae*-infected honeybees fed with a pure sucrose syrup was approximately 1.31 (±0.70) specimens daily per cage (Figs. 5 and 6, SS groups), whereas among *N. ceranae*-uninfected and infected honeybees, whose diet was supplemented with a probiotic, mortality rates were higher, i.e., respectively 1.11 (±0.57) and 1.50 (±0.77) specimens daily per cage (Figs. 4 and 5, L1 group).

Supplementation of honeybee diet with the probiotic and the probiotic + prebiotic (feeding groups: L1, L2, L1 + In, L2 + In) for 9 days before *Nosema* infection (Fig. 6) had the largest impact on honeybee mortality. Inulin present in food together with *L. rhamnosus* promoted the mortality of honeybees associated with the probiotic. However, this prebiotic alone had no visible effect on honeybee death rate (Figs. 5 and 6). An especially high increase in bee mortality was found between the second and the fourth day after microsporidian infection (11th–13th days of the experiment) and reached up to seven specimens per cage (Fig. 6). Over the next few days until the end of the experiment, honeybee mortality was established as being at a constant level, i.e., 2.02 (±0.67) specimens per day per cage (Fig. 6). Generally, we conclude that feeding honeybees with commercial probiotics and probiotic + prebiotic not only does not prevent nosemosis development in bees but may even increase insect vulnerability to infection with *N. ceranae*.

**Fig. 7:**
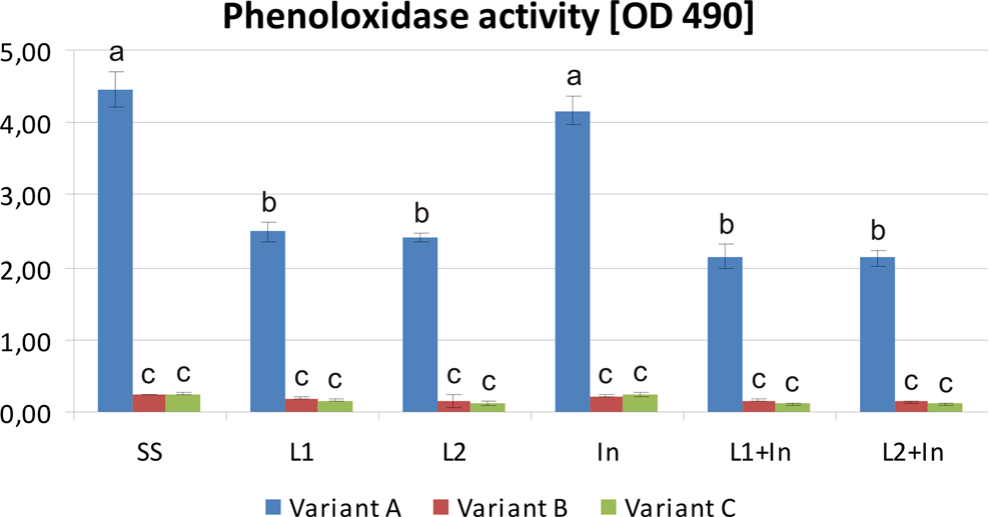
Phenoloxidase activity in the hemolymph of honeybees uninfected and infected with *N. ceranae* fed with commercial probiotics and prebiotics. Variants A, B, and C according to Fig. 1. Phenoloxidase activity was determined using DOPA as a substrate on the basis of melanin formation by measuring absorbance at 490 nm. The diagram demonstrates the enzyme activity after 60 min of incubation. Supplementation among the groups: SS (pure sugar syrup); L1 (1.25 × 10^3^ of *L. rhamnosus* CFU per 1 mL of sugar syrup); L2 (3.75 × 10^3^ of *L. rhamnosus* CFU per 1 mL of sugar syrup); In (inulin 2 μg per 1 mL of sugar syrup). *Error bars* represent standard deviations of data with lowercase letters indicating significant differences (*p* < 0.05)

The prophylactic treatment of humans and different animals with probiotics and prebiotics to enhance their immune defense mechanisms has already been described rather comprehensively (Conway 1989; Ajitha et al. 2004). Nowadays, there is a growing interest in the use of these food supplements for the modulation of honeybee immune systems to prevent and control infectious diseases. The insect immune system relies on innate mechanisms, which in honeybees are greatly reduced in comparison to other insects (Malone and Gatehouse 1998; Hultmark 2003; Evans et al. 2006; Schmid et al. 2008). There are two main categories of these mechanisms, i.e., phagocytosis and the encapsulation of foreign bodies and the antimicrobial activity of immune proteins, e.g., PO, which participates in melanization cascade as the terminal enzyme (Gliński and Buczek 2003). In this study, PO activity (Fig. 7) reached its highest level in the hemolymph of the control honeybees uninfected with *N. ceranae* and fed with a sugar syrup (Fig. 7, variant A, SS group). It was also relatively high in uninfected honeybees fed with a sugar syrup containing the prebiotic (Fig. 7, variants A). Adding the probiotic or the probiotic and prebiotic together to the food of uninfected bees led to a decrease in PO activity, which was approximately two times lower than that in insects fed only with the sugar syrup (Fig. 7, variants A). However, the most negative impact on PO activity was seen in the infection of honeybees with *N. ceranae*. In *Nosema*-infected bees, PO activity was almost 20 times lower than that of uninfected ones, and the absorbance measured at 490 nm after 60 min of incubation was respectively 4.47 and 0.24 for uninfected (Fig. 7, variant A, SS group) and *Nosema*-infected honeybees (Fig. 7, variant B, SS group).

Generally, the infection of honeybees with *N. ceranae* significantly reduced the level of PO activity in the hemolymph. Still, the lowest PO activity was noted when bees were fed for 9 days before infection with a sugar syrup supplemented with *L. rhamnosus* or *L. rhamnosus* together with inulin and reached ~0.14 and ~0.11, respectively (Fig. 7, variants C, groups: L1, L2 and L1 + In, L2 + In). These results clearly indicated the strong inhibition of the honeybees’ PO, not only by microsporidian infection, but also by feeding honeybees with the commercial probiotic and with probiotic in combination with prebiotic. This data suggests that the supplementation of honeybee diets with probiotic or both probiotic and prebiotic is not beneficial for the functioning of honeybee defense systems (Fig. 7).

**Fig. 8:**
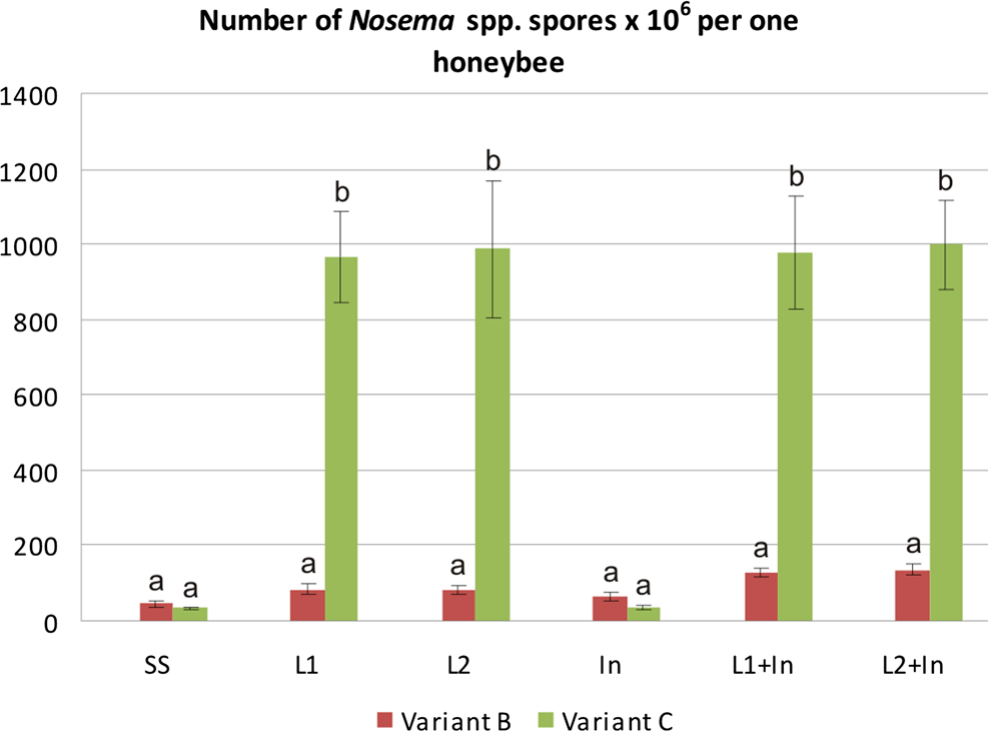
Number of *N. ceranae* spores × 10^6^ per one honeybee fed with commercial probiotics and prebiotics. Variants B and C according to Fig. 1. Supplementation among the groups: SS (pure sugar syrup); L1 (1.25 × 10^3^ of *L. rhamnosus* CFU per 1 mL of sugar syrup); L2 (3.75 × 10^3^ of *L. rhamnosus* CFU per 1 mL of sugar syrup); In (2 μg of inulin per 1 mL of sugar syrup). *Error bars* represent standard deviations of data with lowercase letters indicating significant differences (*p* < 0.05)

Another negative effect of probiotics and/or prebiotics on honeybees was also observed as a rapid and enormous development of nosemosis in the insects. In the control *Nosema*-infected honeybees fed only with sugar syrup, the number of microsporidian spores per honeybee was at ~3.8 × 10^7^ (Fig. 8, groups: SS). The rate of fungi infection, determined by the number of spores, increased up to 25 times in honeybees fed for 9 days before *N. ceranae* infection with a sugar syrup containing the commercial probiotic (Fig. 8, variant C, groups: L1, L2) and a sugar syrup supplemented with both probiotics and prebiotics (Fig. 8, variant C, groups: L1 + In, L2 + In). The infection developed very rapidly and reached levels of 9.7 × 10^8^ and 9.8 × 10^8^ spores per honeybee, in groups L1 and L1 + In, respectively. A similar number of spores per bee, as in groups L1 and L1 + In, was found in L2 and L2 + In groups (Fig. 8). The feeding of honeybees with a sugar syrup supplemented only with the prebiotics before fungi infection did not stimulate the development of the *N. ceranae* infection, as was found in the case of bees fed with a sugar syrup containing probiotic, and the number of microsporidian spores per honeybee was found to be 3.5 × 10^7^ (Fig. 8, variant C, group: In), similarly as in the control groups with ~3.8 × 10^7^ (Fig. 8, group: SS).

We supposed that colonization of honeybees’ intestinal tracts by probiotic microorganisms ought to have inhibited the development of nosemosis, through the competition for binding sites and nutrients, as well as by positive modulation of the immune system. Surprisingly, honeybees fed with a sugar syrup supplemented with commercial probiotic (*L. rhamnosus*) were more susceptible to *N. ceranae* infection and nosemosis development, and the number of microsporidian spores in such bees was very high (Fig. 8). It is possible that lactic acid, produced by multiplying *L. rhamnosus*, could have increased acidity in the bee intestine and/or could have been the cause of degeneration of the gut, and through this, could have initiated favorable conditions for the germination of microsporidian spores and accelerated the infection of epithelial cells with *N. ceranae* (de Graaf et al. 1993; Bradley 2008; Feigenbaum and Naug 2010; Ptaszyńska et al. 2013). Consequently, mortality rates increased among honeybees fed with commercial probiotic containing *L. rhamnosus*. Therefore, preparations containing bacteria identified as probiotics for mammals should not be considered as probiotics for honeybees and possibly for other invertebrates.

Microorganisms selected as commercial probiotics are highly resistant and have a great ability to survive, even in unsuitable environments. Therefore, they can easily proliferate in honeybee intestines and, hence, may exclude natural symbiotic microorganisms. The elimination of honeybees’ natural microbiota can reduce the absorption of nutrients and can lead to the malnutrition of bees. Furthermore, the intensive development of microorganisms, which are non-natural for honeybees, can lead to the degeneration of the peritrophic membranes of the bee intestines which, together with exoskeleton cuticule, are the first lines of insects’ defense against various pathogens. That can be the reason of the increase in the mortality of foragers, as observed in our experiments. In earlier studies (Vásquez and Olofsson 2009; Martinson et al. 2011; Tajabadi et al. 2011, 2013a, b; Pattabhiramaiah et al. 2012; Vásquez et al. 2012; Audisio et al. 2015), different bacterial strains of the genus *Lactobacillus* were isolated from honeybee intestines, meaning these lactobacilli can probably be considered as probiotics, for these ecologically and economically crucial insects.

## Conclusions

The supplementation of honeybee diet with improper probiotics or probiotics and prebiotics can disturb the natural microbiota composition, which is important in maintaining metabolic homeostasis in bee intestines. Furthermore, it can deregulate the immune system and, in consequence, may promote pathogen infections and increase honeybee mortality.
